# Systematic errors in orthology inference and their effects on evolutionary analyses

**DOI:** 10.1016/j.isci.2021.102110

**Published:** 2021-01-28

**Authors:** Paschalis Natsidis, Paschalia Kapli, Philipp H. Schiffer, Maximilian J. Telford

**Affiliations:** 1 Centre for Life's Origins and Evolution, Department of Genetics, Evolution and Ecology, University College London, London WC1E 6BT, UK

**Keywords:** Biological Sciences, Evolutionary Biology, Evolutionary Processes, Evolutionary Mechanisms, Phylogenetics, Phylogeny

## Abstract

The availability of complete sets of genes from many organisms makes it possible to identify genes unique to (or lost from) certain clades. This information is used to reconstruct phylogenetic trees; identify genes involved in the evolution of clade specific novelties; and for phylostratigraphy—identifying ages of genes in a given species. These investigations rely on accurately predicted orthologs. Here we use simulation to produce sets of orthologs that experience no gains or losses. We show that errors in identifying orthologs increase with higher rates of evolution. We use the predicted sets of orthologs, with errors, to reconstruct phylogenetic trees; to count gains and losses; and for phylostratigraphy. Our simulated data, containing information only from errors in orthology prediction, closely recapitulate findings from empirical data. We suggest published downstream analyses must be informed to a large extent by errors in orthology prediction that mimic expected patterns of gene evolution.

## Highlights

• Presence of shared orthologs across species is used for evolutionary analyses
• We simulated realistic sets of orthologs with no gains or losses
• Errors predicting shared orthologs correlate with phylogenetic relationships
• Presence/absence datasets based on errors recapitulate findings from empirical data

## Graphical Abstract

**Figure:**
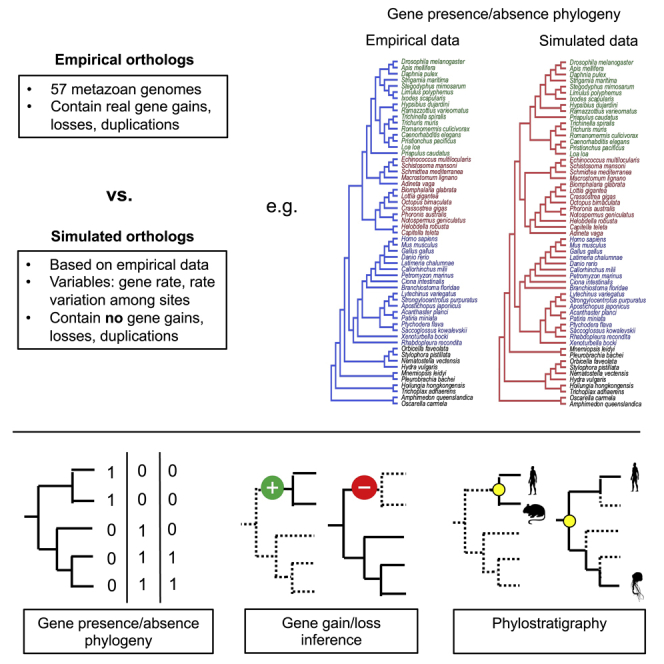

## Introduction

Orthology is a type of homology where the homologous genes originated at a speciation event (Fitch, 1970). The evolution of orthologous genes and the fact that their relationships coincide with species phylogeny have made them key markers in evolutionary biology. Aligned sequences of orthologs have been used to reconstruct species phylogenies for several decades, but the presence or absence of individual orthologs in genomes of different species is also increasingly being used in various ways to understand evolution—something made possible by the largely complete gene sets now available from genome sequencing projects.

New genes that originated in ancestral species and were passed on to the descendants of this ancestor can be used as synapomorphies of these clades. Matrices recording the presence and absence of sets of orthologs across species have been used to give an estimate of relationships that is assumed to be independent of the traditional sequence alignment-based trees (Snel et al., 1999). Gene presence/absence phylogenies of Metazoa have given highly resolved trees showing extraordinary congruence with sequence-alignment-based trees (Ryan et al., 2013; Pisani et al., 2015; Leclére et al., 2019; Pett et al., 2019).

Given a phylogenetic tree, on the other hand, the presence/absence of orthologs in extant taxa can be used to infer gene gain and loss events across their evolutionary history. Such events are being interpreted in the context of origins or loss of key characteristics in those clades. Bursts of gains and losses have been associated with the origins of major animal clades (Fernández and Gabaldón, 2020; Guijarro-Clarke et al., 2020), and a search for genes unique to the Bilateria within Metazoa found 157 candidates that the authors linked to bilaterian morphological novelties such as mesoderm and bilateral symmetry (Heger et al., 2020).

Another use of matrices of gene presence and absence is in phylostratigraphy. Here, genes present in a focal taxon are searched for in the increasingly distant phylogenetic lineages leading to this taxon. In this way it is possible to discover the most distant relatives possessing orthologs and hence to infer the ages of these genes (Domazet-Lošo et al., 2007; Šestak et al., 2013). Sets of genes that may be upregulated in specific developmental stages or structures may have different average phylostratigraphic ages, and this information has been interpreted as implying the evolutionary age of traits such as a larval stage (Wang et al., 2020).

Inferring orthology relationships among thousands of genes that come from distantly related sets of species is a fundamental step in all these studies. Inferring orthologs, however, is an inherently difficult task because the genes in a genome evolve in a complex manner (Glover et al., 2019; Fernández et al., 2020). Orthology inference relies on an initial similarity search to identify, among all pairs of genes in two organisms, those that are sufficiently similar to be potentially homologous (Altenhoff et al., 2019). This step can be difficult if two orthologs have diverged significantly (Jain et al., 2019; Weisman et al., 2020).

Subsequent steps are affected by multiple evolutionary processes: genes are frequently duplicated and lost in different lineages; paralogs produced by duplication can evolve at very different rates; and genes can even be transferred horizontally (Koonin et al., 2001; (Fitzpatrick, 2012); Wickel and Fay-Wei, 2019). Our ability to disentangle the relationships between homologous (but not necessarily orthologous) genes is further hampered by the heterogeneities that affect the reconstruction of gene phylogenies such as heterogeneities in evolutionary rates or compositional bias (Kapli et al., 2020).

The three important downstream uses of orthologs we outlined above (presence-absence phylogenies; plotting gene gains/losses across a phylogeny; and phylostratigraphy) must be affected by misidentification of orthologs, but there has been relatively little consideration in these studies of the error rates of the methods used to predict orthologs.

Previous work has shown that specific attributes of genes—especially higher rates of evolution—can make ortholog identification more difficult. Elhaik et al. (2006) used simulations to show that it became increasingly difficult to detect homology the faster genes evolved. Luz and Vingron (2006) also showed genes whose orthologs are found widely among a set of taxa tend to be slower evolving. Moyers and Zhang (2014, 2017) have, like us, used simulation of gene evolution followed by a phylostratigraphic analysis and showed that faster evolving genes appeared younger than they should—i.e that orthologs of faster genes are less likely to be detected in more distant relatives. Recently, Weisman et al. (2020) showed that supposedly lineage-specific genes within the *Drosophila* and *Saccharomyces* genera have undetected homologs outside their respective lineages. Martin-Durán et al. made similar observations of supposedly lineage restricted genes of Platyhelminthes (Martín-Durán et al., 2017), which could be found in more distant taxa when less stringent searches were conducted.

It certainly makes intuitive sense that, as genes become more distinct, our ability to detect homology between them will diminish. It seems less obvious what effect inaccuracies resulting from such problems in orthology prediction will have on any downstream analyses: are they random with neutral effects or could there be systematic errors in orthology prediction that produce strongly supported results? Part of the difficulty in answering these questions is that, when using empirical data, we do not know the underlying truth.

**Figure 1:**
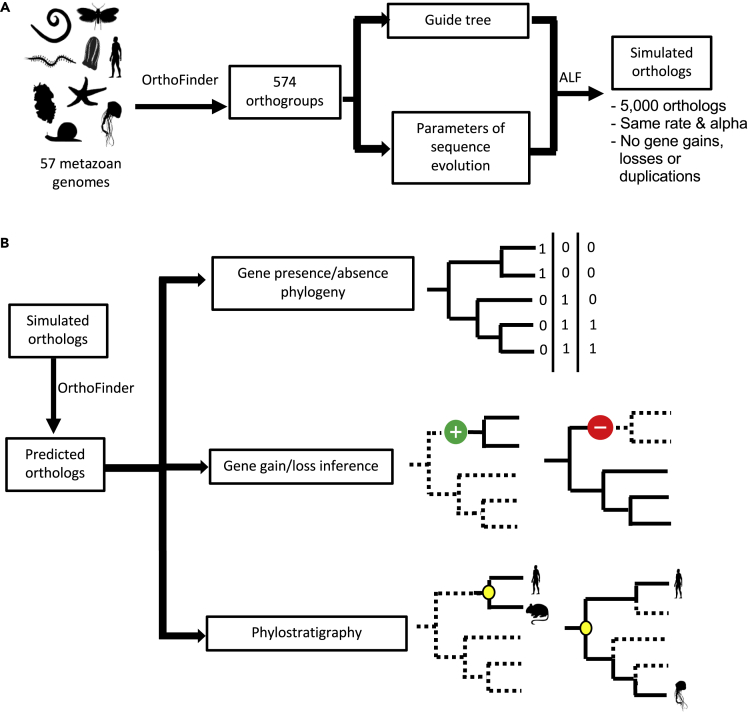
Workflow diagram (A) We used information from 574 metazoan orthologs from 57 genomes to infer realistic parameters of sequence evolution to inform our simulations. Two hundred sets of 5,000 orthologs were simulated according to the empirically derived parameters and a fixed tree topology without any gene gains, losses, or duplications. (B) Orthology relationships among each of the simulated orthologs were inferred using OrthoFinder. These results were used in three different downstream analyses to understand the impact of orthology prediction error: gene presence/absence phylogeny; gene gain/loss inference; and phylostratigraphy.

To overcome this limitation, we have, like Moyers and Zhang (2014, 2017), simulated the evolution of sets of orthologous genes along a tree. Our simulations were conducted using a relatively large phylogeny that is based on the metazoan tree. We derive realistic parameters from empirical data from 57 metazoan species (Figure 1A) to inform our sequence simulation. We used our sets of simulated orthologs to examine the relationship between the frequency of orthology prediction errors and two important aspects of sequence evolution: (1) substitution rate and (2) the variance of rates across sites within a gene. Finally, we have explored the effects of these errors on gene presence/absence phylogenies; mapping gene gains and losses on a phylogeny; and phylostratigraphy (Figure 1B).

## Results and discussion

### Effects of gene rate and between-site rate heterogeneity on OrthoFinder accuracy

**Figure 2:**
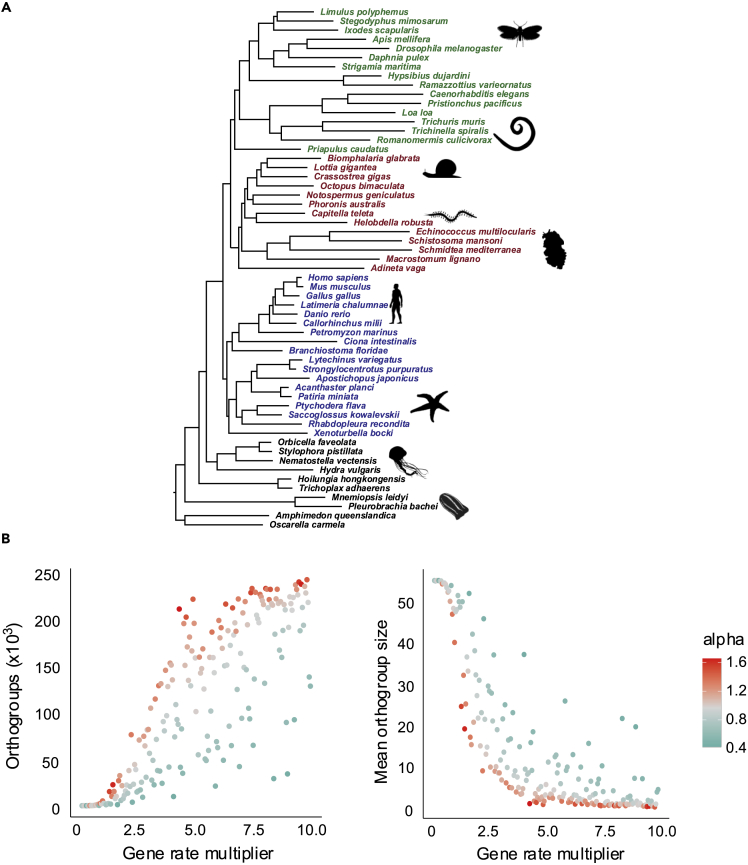
Errors in orthology prediction among simulated orthologs are more frequent with faster genes and with higher alphas (A) The guide tree under which the orthologs evolved in our simulations. Branch lengths were estimated based on the concatenated set of 574 orthogroups using the LG + F + G + C60 model. Each simulation involved the evolution of 5,000 orthologs along a scaled version of this guide tree, where all branch lengths were multiplied by a scalar ranging from 0.2x to 10x. Green: Ecdysozoa, Red: Lophotrochozoa, Blue: Deuterostomia, Black: Non-Bilateria. (B) Number of orthogroups inferred from each of the 200 simulation replicates plotted according to rate of evolution and alpha. An accurate inference would contain 5,000 orthogroups (left). Mean orthogroup size inferred from each of the 200 simulation replicates plotted according to rate of evolution and with different alphas (right). An accurate inference would show orthogroups containing 57 species. Higher orthogroup sizes indicate more errors. In simulations with small gene rate multipliers (corresponding to slow-evolving genes) orthology inference was successful in recovering 5,000 orthologs with the correct mean size of 57 genes. With larger gene-rate multipliers, orthology inference erroneously inferred more and smaller orthogroups. Higher alphas (less between-site rate heterogeneity) resulted in more errors in orthology inference.

We used a fixed phylogeny based on the topology relating 57 metazoan taxa (Figure 2A) and used 574 orthologs predicted from these species to make estimates of several parameters of sequence evolution. Using these parameters estimated from empirical data, we ran 200 simulations, each of which produced 5,000 sets of orthologs present in all the 57 species. For each of the 200 sets of proteins, we used a simulation-specific gene rate multiplier to simulate genes evolving at different rates and a simulation-specific alpha parameter to simulate genes with various degrees of site rate heterogeneity. We ran OrthoFinder (Emms and Kelly, 2015) using default settings on all 200 sets of 5,000 genes. With perfect orthology prediction, we would expect to recover exactly 5,000 orthogroups and each orthogroup would contain exactly 57 genes, one for every species. Any divergence from these numbers will be due to orthology inference errors.

Figure 2B shows the relationship between both the gene rate multiplier and the alpha parameter and the numbers of predicted orthogroups and mean orthogroup sizes. With small gene rate multipliers, representing slowly evolving genes, OrthoFinder was very successful in recovering the correct number of orthogroups. As the gene rate multiplier increases, however, we observe increasing numbers of errors—the predicted number of orthogroups becomes higher than 5,000. In simulations with the highest gene rate multipliers we see as many as 250,000 orthogroups, and mean orthogroup size is much smaller than the true size of 57 genes/orthogroup.

We wanted to see whether the relationship between rate of evolution within an orthogroup and the number of species included in that orthogroup is also reflected in empirical data. To control for phylogenetic distance, we considered four pairs of species (Figure S1), and for each pair we considered all orthogroups containing both species. For each of these orthogroups, we measured the patristic distance between the pair of species and compared this distance (which is a relative rate) with the total number of species represented in that orthogroup. As with our simulated data, we found an inverse correlation between the relative rate of evolution of a gene and the number of species in the orthogroup containing that gene (Figure S1), suggesting that the relationship between rate and frequency of errors in orthology prediction we observe in simulated data may also be true of real data.

The alpha parameter for rate variation among sites has an independent effect on the frequency of error. For a given gene rate multiplier, higher values of alpha (less between-site rate heterogeneity) lead to more errors. The higher frequency of errors with increasing gene rates is not unexpected. As orthologs become more divergent, it becomes more difficult to determine their homology. The effect of the higher alpha parameter on our ability to infer orthologs correctly is less easily explained, however, because this affects only the distribution of rates across sites (higher alpha parameters have more uniform rates) but not the mean rate. The low alphas (more skewed rates across sites) mean that there are many slowly evolving sites and a few fast-evolving sites. We speculate that the presence of sufficient numbers of slow-evolving sites would permit the similarity searching stage of orthology prediction to find regions that are similar enough for the genes to be considered as homologs. For genes with higher alphas (a more even distribution of intermediate rates) if the rate across the whole gene is high enough, then the similarity search may fail to find any regions with sufficient similarity to warrant further consideration by the Diamond BLAST algorithm (Buchfink et al., 2015).

### Gene presence/absence phylogenies are informed by errors in orthology inference

The availability of well characterized metazoan genomes has allowed the presence or absence of orthologous genes in different species to be used as characters to reconstruct phylogenetic relationships (e.g. metazoan phyla [Ryan et al., 2013; Pisani et al., 2015; Leclére et al., 2019; Pett et al., 2019] and Insecta [Rosenfeld et al., 2016; 2017]). The assumption underlying these studies is that the 1s and 0s of the matrix represent real presences and absences of genes within genomes and hence can reveal gains and losses of genes through evolution. These presence/absence phylogenies are highly congruent with sequence-based metazoan phylogenies (Cannon et al., 2016; Marlétaz et al., 2019; Philippe et al., 2019; Laumer et al., 2019). It is not clear, however, whether the phylogenetic signal contained in the gene presence/absence matrices might be affected by orthology inference errors and how these errors might influence the resulting tree.

The inferred sets of orthologs from our simulation experiments each constitute a matrix of presence and absence of orthologs suitable for phylogeny reconstruction. We wanted to know what effect the errors we observe (especially with faster evolving genes (Figure 2B) and higher alphas) might have on our ability to reconstruct gene presence/absence phylogenies. Because we simulated without any gene gain, loss, or duplications, any phylogenetic information in the matrix will come solely from orthology prediction errors.

**Figure 3:**
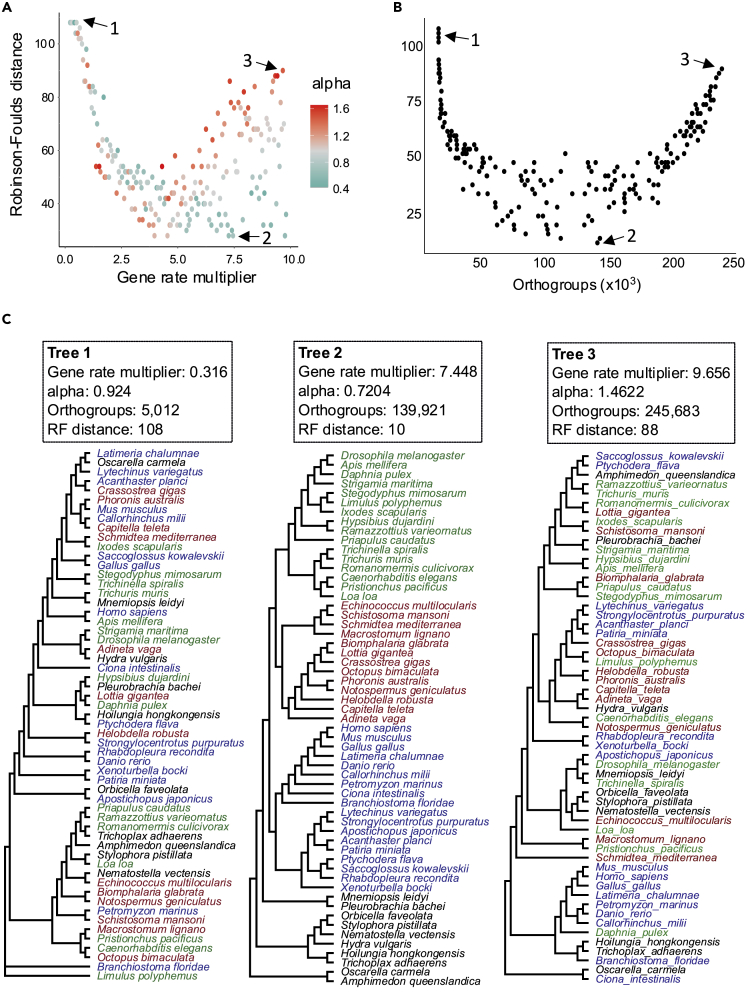
Gene presence/absence phylogenies benefit from errors in orthology inference (A) Relationship between gene evolution rate on the accuracy of trees reconstructed from the per-species presence//absence matrix for each simulation. Accuracy is calculated using the Robinson-Foulds distance (RF) between the true tree and the reconstructed tree. In simulations of slow-evolving genes (few orthology inference errors) the corresponding presence/absence trees are very poor (High RF). For faster-evolving simulations (more orthology inference errors) the trees become much more accurate. For slower genes a higher alpha gives better trees. As the rate increases, a lower alpha results in superior trees. The values corresponding to the trees (1,2,3) shown in part C are indicated by arrows. (B) The most accurate trees correspond to an intermediate level of error as measured by the number of inferred orthogroups. With very low and very high error rates the trees are very poor. The values corresponding to the trees (1,2,3) shown in part C are indicated by arrows. (C) Examples of trees reconstructed using matrices of gene presence/absence based on slow-, intermediate-, and fast-evolving simulations. The trees correspond to the points indicated by arrows in Figure parts A and B. The parameters used in the three simulations are indicated in the boxes. Green species are ecdysozoans, brown species are lophotrochozoans, and blue species are deuterostomes.

For each simulation we used the resulting gene presence/absence matrix to build a phylogenetic tree using RAxML (Stamatakis, 2014) with the BINGAMMA model as appropriate for the evolution of binary characters. We used the Robinson-Foulds (RF) distance to measure the difference between the true tree relating the taxa (the simulation guide tree) and each tree built using the orthologs inferred from our simulated data (Figure 3A). RF measures the number of splits that differ between two trees: RF is 0 if trees are identical.

Slowly evolving genes, where most or all orthologs were correctly predicted, naturally contained little or no phylogenetic information, giving an RF over 100. As we considered faster-evolving sets of genes, we saw the appearance of phylogenetic structure in the trees and a large decrease in RF values. The best trees (RF ∼10) were observed in simulations with higher rates but when alphas are low. Higher rates with higher alphas perform poorly. The best trees actually correspond to an intermediate rate of orthology inference error as can be seen in Figure 3B. Examples of trees from the extremes of this distribution (very few or very many errors) illustrate the poor estimates of phylogeny compared with the best trees we observe at intermediate levels of error (Figure 3C). Importantly, the orthology errors caused by high substitution rates and small alphas are far from random; they contain information that accurately reflects the underlying species relationships.

**Figure 4:**
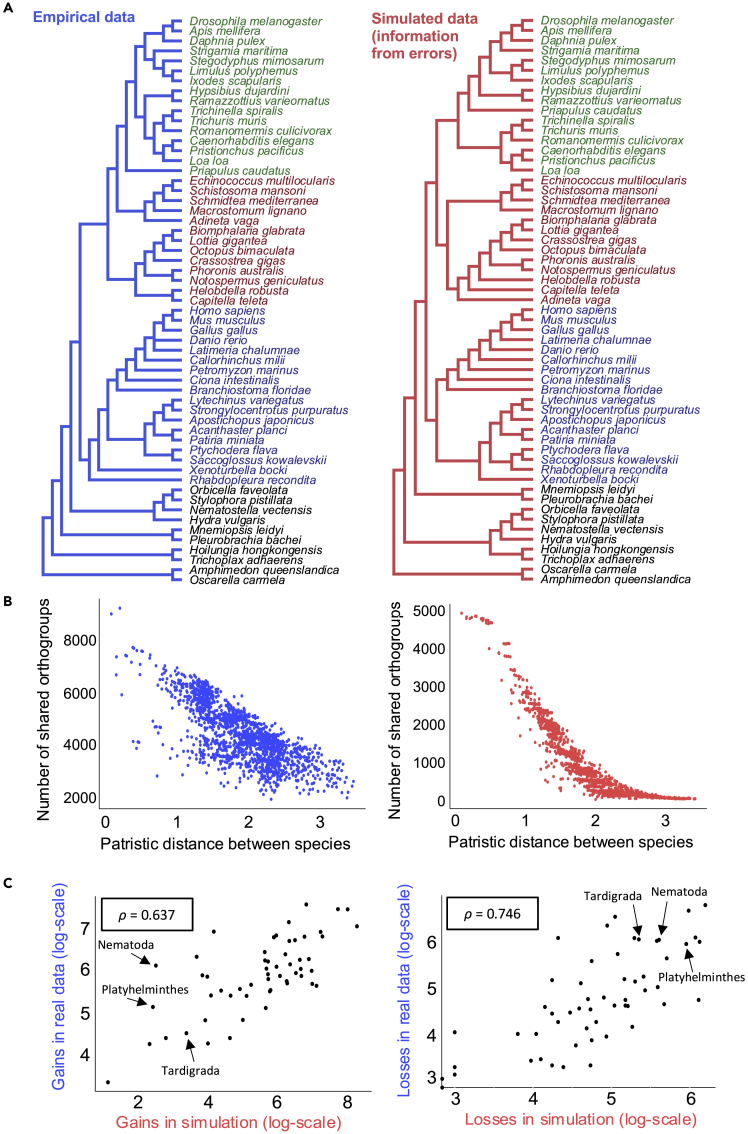
Downstream analyses based on orthology prediction errors in simulated data closely resemble the results from real data (A) A phylogenetic tree reconstructed using the gene presence/absence matrix from real data (left/blue) closely resembles the tree based on orthology prediction errors from simulated data (right/red). Green species are ecdysozoans, brown species are lophotrochozoans, and blue species are deuterostomes. (B) The number of orthogroups shared between pairs of species correlates with the patristic distance between them for orthology predictions based on both real data (left/blue) and for simulated data for which all information results from errors (right/red). (C) Comparison of numbers of gene gains and losses in each node of the guide tree estimated from real (y axis/blue) and simulated (x axis/red) data. Numbers of gene gains in each node (left) and gene losses (right) are strongly correlated between simulated and real data. Each dot represents an internal node of the guide tree. The values for the nodes leading to the fast-evolving tardigrades, platyhelminths, and nematodes are indicated. The correlation coefficient ρ was calculated using Spearman's rank test.

To compare our best trees from simulated data with a tree built using real presence/absence data, we built a gene presence/absence matrix using orthogroups predicted using OrthoFinder on our sets of genes from the same 57 species and reconstructed a phylogeny (Figure 4A). We compared this real presence/absence tree with the best-scoring presence/absence tree from our simulations (simulation with gene rate multiplier = 7.448, alpha = 0.7204, RF = 10). We found that the tree generated using real data is highly congruent with the best tree from our simulations (resulting purely from orthology errors) (Figure 4A). Much of the phylogenetic signal in the real gene presence/absence matrix may be derived from errors in orthology inference rather than from real gene gains and losses.

At least for datasets with intermediate levels of orthology inference errors, the set of orthologs that any given pair of species have in common reflects their phylogenetic relationships; we infer that the number of orthologs in common to any given pair of species would therefore be related to their evolutionary distance. To show this for all possible pairs of species in the guide tree (Figure 2A), we plotted the patristic distance between them (the sum of branch lengths separating them) against the number of inferred orthogroups they share. We did this both for the real data and for the simulation that had resulted in the most accurate tree. For both real and simulated data (Figure 4B) we see a strong negative correlation between the number of shared orthologs and the evolutionary distance between species. For the real data this relationship is likely to come from a mixture of real gains and losses and rate-related errors.

Our results show that the errors in orthology are far from random but are strongly correlated with phylogenetic relationships of the species in question and the degree of similarity among their orthologs. Faster-evolving genes are less likely to be correctly grouped as orthologs. We find that genes in pairs of species that are evolutionarily distant are also less likely to be correctly identified as orthologs. These errors are exaggerated when the alpha parameter for site-specific rate variation is sufficiently large.

### Numbers of gene gains and losses are systematically overestimated due to orthology inference errors

The ability to work with complete sets of genes within genomes has prompted efforts to infer the series of gene gains and losses that occurred along the internal branches of the evolutionary tree relating a given set of species (Fernández and Gabaldón, 2020; Guijarro-Clarke et al., 2020). This approach is seen as a way to uncover possible genomic correlates of important phenotypic transitions in evolutionary history: the evolution of clade-specific novelties; losses of certain morphological characteristics; or appearance of certain embryological characters (Wang et al., 2020). One inference from recent work is that the evolution of the metazoan gene repertoire has been driven to a great extent by gene loss events (Fernández and Gabaldón, 2020), with losses especially prominent in the branches leading to some fast-evolving animal phyla (Nematoda, Tardigrada, Platyhelminthes) (Guijarro-Clarke et al., 2020). We have used an equivalent analysis to map gains and losses of genes onto our guide tree (Swofford, 2013). Using our presence/absence matrix derived from real data, we counted gains and losses at different nodes of the tree reconstructed. We compared these empirical findings with results from one of our matrices derived from simulated data.

For each internal branch of the guide tree, we compared the number of gene gains and losses inferred at each node using real data and using the simulated data with the lowest RF score (see Figure 3). We observed a strong correlation (correlations: gains ρ = 0.637; losses ρ = 0.746. Figure 4C); the similarity between real data and our simulations suggests that some of the apparent gene gains and losses in analyses of real data are likely to be due to systematic errors during orthology inference related to the distance between taxa.

### Apparent gene ages in phylostratigraphy analyses are correlated with rates and phylogenetic distance

Phylostratigraphic analyses estimate the ages of each member of the set of genes in a focal species by looking for their orthologs in increasingly distantly related sister clades. The most distant outgroup species or clade in which a homolog is found defines the age of the gene. Simulations have been used previously to assess the accuracy of phylostratigraphy and have shown an inverse correlation between rate of evolution and apparent gene age (Moyers and Zhang, 2014, 2017; Domazet-Lošo et al., 2017).

For each of our simulated sets of orthologs, we considered *Homo sapiens* as the focal species and, for each orthogroup containing a human gene, we found the most distant sister clade also contained in the orthogroup and assigned a corresponding age value to each gene (Figure S2A). For each simulation we calculated the average age value of the 5,000 human genes to give an Average Gene Age (AGA). We plotted AGA in relation to the gene rate multiplier and the alpha of each simulation (Figure S2B). For simulations of slowly evolving genes, the AGA is close to 1 (most/all genes originating in the metazoan ancestor). For simulations with faster evolving genes, the AGA steadily increases, meaning that the 5,000 human genes appear to be younger than they are.

### Conclusions

Sets of predicted orthologs are being used in different ways for several important evolutionary analyses, but correct orthology identification is known to be difficult (Glover et al., 2019; Fernández et al., 2020), especially between more distantly related species—genes inevitably become more distinct as they diverge (Jain et al., 2019; Weisman et al., 2020). As has been pointed out (Emms and Kelly, 2020), whereas most methods have relatively good precision, the persistent problem in orthology inference appears is low recall, meaning that genes are often missing from orthogroups, or orthogroups are fragmented. An implicit assumption of the downstream analyses of sets of orthologs is that, although errors in identifying orthologs are to be expected, (1) they should not be frequent, and (2) there should be no systematic biases in the distribution of the errors that would be interpreted as signal in subsequent steps.

We have shown that errors can be frequent and that they are not randomly distributed when we consider realistic simulated sets of orthologs. We suggest that this problem is also very likely to affect real data. Although the likely artificial enhancement of the phylogenetic signal might optimistically be seen as a benefit in the case of presence/absence phylogenies, conclusions drawn concerning the gains and losses of genes on a phylogeny may well have been based on a misleading signal derived from the systematic biases we have identified. Phylostratigraphic analyses will be similarly affected, with fast-evolving genes appearing to be younger than their real age, as has been pointed out previously (Moyers and Zhang, 2014, 2017). Although not explicitly tested here, other work exploiting predicted co-occurrence of orthologs, such as inferring gene-gene functional associations through phylogenetic profiling, are likely to be affected by the correlation between rate of evolution and rate of error. Apparent co-occurrence or co-absence may in some cases reflect similar rates of evolution.

It is not immediately obvious how to separate the signal derived from gains and losses that are to be expected of a real evolutionary process from apparent gains and losses due to errors in orthology inference. The apparent gains and losses that our simulations predict will follow a very similar pattern of distribution to that expected of real events. This problem is compounded by the fact that we cannot know the true pattern of gains and losses, making it difficult to estimate the size of the problem.

Our simulations, although missing important aspects of the process of gene evolution, could be used to derive an approximate null expectation of the degree of error that might be subtracted from the total signal. Branches with numbers of gains and losses strikingly in excess of the null expectation are likely to represent a real signal indicating a spike in gene gains or losses. Ultimately, however, extra steps to correct for errors correlated with evolutionary distance are required; the large effect of different values of alpha on the frequency of error should be an interesting new avenue of research. We note that, although we have based our experiments on the use of the popular OrthoFinder software, we expect that use of alternative orthology prediction software would give similar results.

We have shown that each of the results we derived from simulated data can mirror observations from empirical data. Although some of the evolutionary signals found in the sets of orthologs derived from empirical data must represent real events of gene gain and loss, our results suggest that this signal is likely to be supplemented to an unknown degree by the systematic errors we have described.

### Limitations of the study

The effects of different parameters of orthology inference (inflation parameter, e-value cut off, different algorithms/software) were not explored in the present study. The presence/absence trees for simulated data were reconstructed using different software than was used for real data. This was to accommodate the different expectations we had as regard missing data for the two datasets that necessitated different models. The comparison is purely illustrative, however, and we do not anticipate that re-running with different software and inappropriate models would change our results.

### Resource availability

#### Lead contact

Further information and requests for resources should be directed to and will be fulfilled by the Lead Contact, Maximilian J. Telford (m.telford@ucl.ac.ukl).

#### Materials availability

This study did not generate new unique reagents.

#### Data and code availability

The datasets and code generated during this study are available at https://github.com/MaxTelford/Gainsandlosses.

## Methods

All methods can be found in the accompanying Transparent Methods supplemental file.

## Supplemental information

Document S1. Transparent methods and figures S1 and S2
