# Supplementary material for: Systematic errors in orthology inference and their effects on evolutionary analyses
Source: iScience. 2021 Jan 28;24(2):102110. doi: 10.1016/j.isci.2021.102110 (PMC7892920; doi:10.1016/j.isci.2021.102110)
Supplement: Document S1. Transparent methods and figures S1 and S2 [file mmc1.pdf]

iScience, Volume *24*

Supplemental Information

Systematic errors in orthology inference and their effects on evolutionary analyses

Paschalis Natsidis, Paschalia Kapli, Philipp H. Schiffer, and Maximilian J. Telford

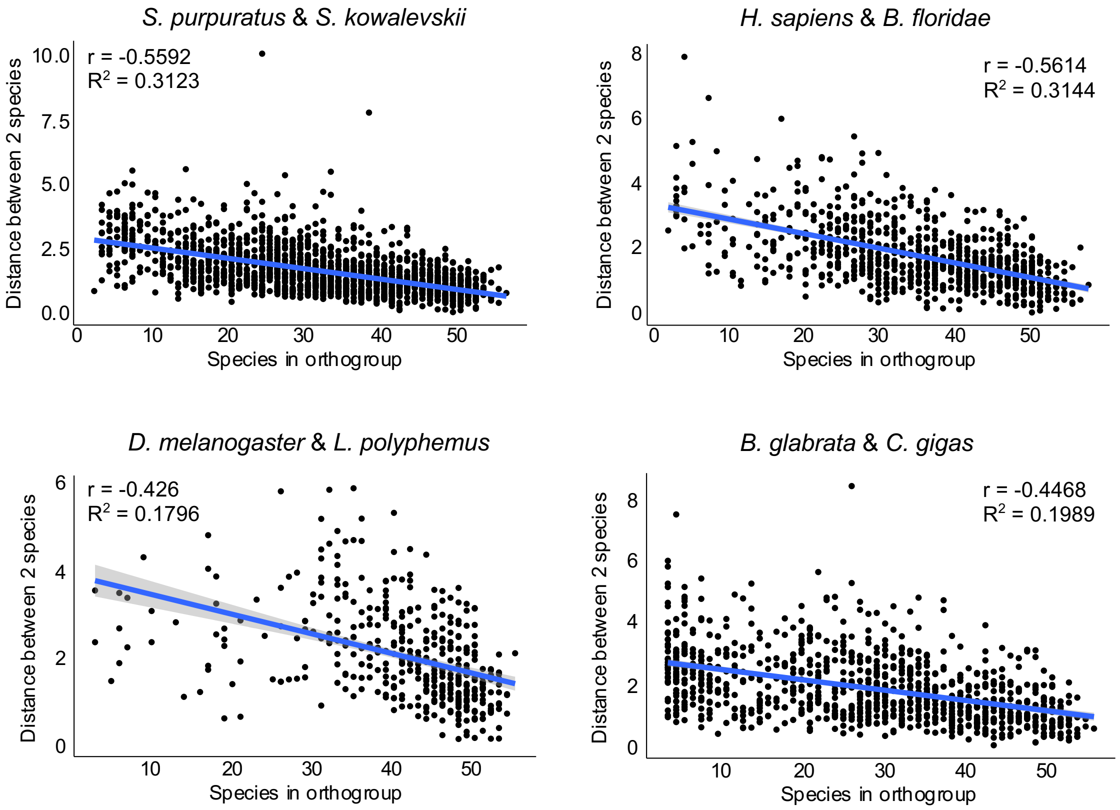

Figure S1. Faster-evolving genes are found in orthogroups with fewer taxa for empirical data, Related to Figure 2.
Each dot represents an orthogroup in which both species of the pair indicated are present. The data are from real genomes. The x axis shows the total number of different species present in the orthogroup and the y axis shows the patristic distance between the two species of the pair in the orthogroup tree (a measure of the rate of the gene). There is a small negative correlation trend between the two variables as shown by the Pearson's correlation coefficient (*r*) and the adjusted R-squared of linear regression analysis (R^2).

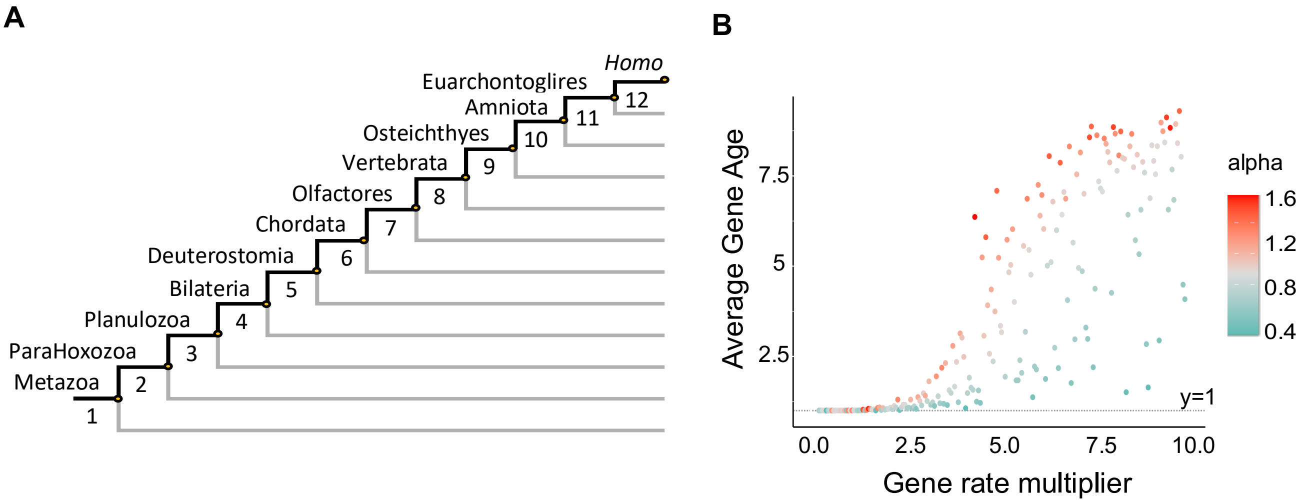

Figure S2. Fast-evolving genes appear younger than they are in phylostratigraphic analysis, Related to Figure 2.

a. The phylostrata used to calculate the age of each human gene in each simulation replicate. The numbers at each node were used to calculate the Average Gene Age (AGA).

b. Average gene age scores for the 200 simulation replicates. The score represents the average phylostratum value for each of the 5,000 human genes in the simulation. The expected score with no orthology inference errors would be 1.

Transparent Methods

Extracting simulation parameters from empirical data and simulating orthologs.

We chose to simulate the evolution of sets of orthologous genes across a tree based on the relationships among metazoan phyla. We collected the set of predicted proteins from 57 well-characterised metazoan genomes selected to give a broad representation of the different metazoan clades (10 non-Bilateria, 9 Xenambulacraria, 9 Chordata, 13 Lophotrochozoa, 16 Ecdysozoa) and exhibiting different rates of evolution.

We used a consensus of the current status of metazoan phylogeny [16-19] as a guide tree for our simulations of gene evolution. To get realistic estimates of its branch lengths, we ran OrthoFinder v2.3.1 (Emms and Kelly, 2015) with DIAMOND v0.9.24.125 (Buchfink et al., 2015) using our 57 genomes and selected 574 near-global orthogroups (minimum 80% taxon occupancy). We then aligned with MAFFT v7.455 (Katoh et al., 2002), trimmed with BMGE v1.12 (Criscuolo and Gribaldo, 2010) and concatenated into an alignment of 210,516 amino acids. The branch lengths of the guide tree were then calculated using IQ-tree v1.6.12 (Nguyen et al., 2015) with the LG+F+G+C60 model and our concatenated alignment.

We used the 574 single-copy orthogroups and the fixed guide tree (iqtree -g command) to estimate the following parameters of the LG model of sequence evolution: amino-acid frequencies, gene tree lengths (sum of branch lengths) and alpha parameters for rate variation among sites. These sets of parameters were subsequently fitted into distributions (Dirichlet for amino acid frequencies, normal for alphas), that were later used to provide realistic parameter values for the simulations. The tree lengths were used to define guide tree multipliers (gene rate multipliers) in order to simulate slower and faster evolving genes. The multipliers obtained from the 574 orthogroups ranged from 0.2x to 3x, but we extended the range up to 10x in order to capture genes with evolutionary rates that were presumably missed during our stringent approach for selecting orthogroups.

Running simulations of sets orthologous genes.

We performed our simulation experiments using ALF (Dalquen et al., 2011) under the guide tree and using parameter values derived from real data as described above that were provided in a replicate-specific configuration file. We created 200 ALF configuration files with settings for the guide tree lengths (guide tree in Fig. 2 with all branch lengths multiplied by a scalar between 0.2x and 10x), amino-acid frequencies and alpha parameter for rate variation among sites were chosen at random from the empirically derived distributions described above. Each configuration file produced one simulation repeat. Each repeat was run with 5,000 starting genes, 100 possible amino-acid frequency states and a single alpha value of the gamma distribution to model rate variation among sites. Each of the 5,000 genes was evolved along the guide tree according to the LG matrix exchangeabilities and independent from other genes. We did not allow for any gene losses or duplications to occur during the simulated gene evolution. As a result, at the end of each simulation we had 5,000 sets of orthologs present in a single copy in all 57 species.

Orthology inference.

We inferred orthology relationships among the simulated sets of orthologs using OrthoFinder v2.3.1 (Emms and Kelly, 2015) using the default settings. We did one orthology inference per simulation repeat. From the OrthoFinder output, we counted the number of resulting orthogroups and the mean orthogroup size. Since each simulation repeat was run with a specific guide tree length and a specific alpha parameter for rate variation among sites, we were able to correlate these two parameters with the number of orthology errors that we observed.

Gene presence/absence phylogeny inference.

We converted the Orthogroups.GeneCounts.csv matrix from the OrthoFinder result to a gene presence/absence binary alignment using a custom script (orthocounts2bin). This script creates a FASTA or PHYLIP alignment from the gene count matrix where every non-zero character is coded as 1 and every zero character is coded as 0.

Since the gene count matrix does not contain information for the unassigned orthologs (singletons, ‘orphan’ genes), we added these to the gene presence/absence binary alignment using a custom script. The resulting per-species ortholog presence/absence matrix (singletons included) was used to infer gene presence absence phylogenies using RAxML v8.2.12 (Stamatakis, 2014) using the ‘-m BINGAMMA’ model. No ascertainment bias correction was used since singletons are present in the alignment. We also reconstructed a tree using the gene presence/absence information in the real sets

of genes from the 57 species. We used MrBayes v3.2.6 (Ronquist and Huelsenbeck, 2003) with the F81-like model for binary data and using the ascertainment bias corrections ‘nosingletonpresence` and `noabsencesites`.

Gene gain and loss inference.
We used a parsimony optimisation approach in PAUP* v4.0a (Swofford, 2013) to infer gene gain and loss events in each internal node of the guide tree. We converted the 200 gene presence/absence matrices into nexus scripts suitable for PAUP* input using a custom python script. We then ran the data matrix with the script in PAUP* for each of the 200 simulations and parsed the PAUP* output to infer the number of gene gain and loss events that occurred on each internal node. We did the same for a per-species ortholog presence/absence matrix derived from the real sets of genes from the 57 species.

Phylostratigraphy analysis.
We used the OrthoFinder results from our 200 simulated sets of proteomes to examine the effect of orthology error on phylostratigraphic analyses of gene age. We chose the human as focal species, and looked at the 5,000 orthogroups that contained a human gene. We assigned an age to each of these orthogroups based on the species it contained, according to Fig. 6A. Each simulation received an average gene age score that is the average age over all orthogroups that contained a human gene (Fig. 6B).

Supplemental References

Criscuolo, A. and Gribaldo, S. (2010). BMGE (Block Mapping and Gathering with Entropy): a new software for selection of phylogenetic informative regions from multiple sequence alignments. BMC Evol. Biol. *10,* 210.

Dalquien, D., Anisimova, M., Gonnet, G. H. and Dessimoz, C. (2011). ALF – a simulator framework for genome evolution. Mol. Biol. Evol. *29,* 1115-1123.

Katoh, K., Misawa, K., Kuma, K.-I. and Miyata, T. (2002). MAFFT: a novel method for rapid multiple sequence alignment based on fast Fourier transform. Nucleic Acids Res. *30,* 3059-3066.

Nguyen, L., Schmidt, H. A., von Haeseler, A. and Bui, Q. M. (2015) IQ-tree: A fast and effective stochastic algorithms for estimating maximum-likelihood phylogenies. Mol. Biol. Evol. *32,* 268-274.

Ronquist, F. and Huelsenbeck, J. P. (2003). MrBayes 3: Bayesian phylogenetic inference under mixture models. Bioinformatics. *19,* 1572-1574.
